# Prospects for Developing Odour Baits To Control *Glossina fuscipes* spp., the Major Vector of Human African Trypanosomiasis

**DOI:** 10.1371/journal.pntd.0000435

**Published:** 2009-05-12

**Authors:** Maurice O. Omolo, Ahmed Hassanali, Serge Mpiana, Johan Esterhuizen, Jenny Lindh, Mike J. Lehane, Philippe Solano, Jean Baptiste Rayaisse, Glyn A. Vale, Steve J. Torr, Inaki Tirados

**Affiliations:** 1 International Center for Insect Physiology and Ecology, ICIPE, Nairobi, Kenya; 2 Masinde Muliro University of Science & Technology, Kakamega, Kenya; 3 Labovet, Kinshasa, Democratic Republic of Congo; 4 Liverpool School of Tropical Medicine, Liverpool, United Kingdom; 5 Institut de Recherche pour le Développement (IRD), UMR 177 IRD-CIRAD, Montpellier, France; 6 Centre International de Recherche-Développement sur l'Elevage en Zone Subhumide (CIRDES), Bobo Dioulasso, Burkina Faso; 7 Natural Resources Institute, University of Greenwich, Chatham, United Kingdom; Yale University School of Medicine, United States of America

## Abstract

We are attempting to develop cost-effective control methods for the important vector of sleeping sickness, *Glossina fuscipes* spp. Responses of the tsetse flies *Glossina fuscipes fuscipes* (in Kenya) and *G. f. quanzensis* (in Democratic Republic of Congo) to natural host odours are reported. Arrangements of electric nets were used to assess the effect of cattle-, human- and pig-odour on (1) the numbers of tsetse attracted to the odour source and (2) the proportion of flies that landed on a black target (1×1 m). In addition responses to monitor lizard (*Varanus niloticus*) were assessed in Kenya. The effects of all four odours on the proportion of tsetse that entered a biconical trap were also determined. Sources of natural host odour were produced by placing live hosts in a tent or metal hut (volumes≈16 m^3^) from which the air was exhausted at ∼2000 L/min. Odours from cattle, pigs and humans had no significant effect on attraction of *G. f. fuscipes* but lizard odour doubled the catch (*P*<0.05). Similarly, mammalian odours had no significant effect on landing or trap entry whereas lizard odour increased these responses significantly: landing responses increased significantly by 22% for males and 10% for females; the increase in trap efficiency was relatively slight (5–10%) and not always significant. For *G. f. quanzensis*, only pig odour had a consistent effect, doubling the catch of females attracted to the source and increasing the landing response for females by ∼15%. Dispensing CO_2_ at doses equivalent to natural hosts suggested that the response of *G. f. fuscipes* to lizard odour was not due to CO_2_. For *G. f. quanzensis*, pig odour and CO_2_ attracted similar numbers of tsetse, but CO_2_ had no material effect on the landing response. The results suggest that identifying kairomones present in lizard odour for *G. f. fuscipes* and pig odour for *G. f. quanzensis* may improve the performance of targets for controlling these species.

## Author Summary

Human African Trypanosomiasis (sleeping sickness) remains a serious threat to many of the poorest people in Africa. The trypanosomes causing the disease are transmitted by tsetse flies. There are no vaccines or prophylactic drugs to prevent people from contracting the disease. So the disease is dealt with after it has been contracted using often ineffective curative drugs with unpleasant and sometimes fatal side effects. Prospects for the development of effective vaccines or prophylactic drugs are poor. One certain means of preventing disease transmission is to remove tsetse flies, either at a local level (e.g., a group of villages) or regionally (covering large parts of a country or region). However, a major problem is the cost and logistical difficulty of implementing such fly control programmes. To overcome this, we are trying to develop cost-effective insecticide-treated targets by identifying chemicals that will increase the numbers of tsetse that will be lured to a target and killed. Here we show that two major vectors, *G. f. fuscipes* and *G. f. quanzensis*, are attracted to the odour of monitor lizards and pigs, respectively. This opens the way for further work to identify the attractants present in these natural odours which can be simply and cheaply incorporated into targets to reduce the cost of control.

## Introduction

Between 1931 and 1961, the annual number of recorded Human African Trypanosomiasis (HAT) cases was reduced by >90%, from >60,000 reported cases/year to <5000 cases/year, through the systematic screening and treatment of millions of individuals across sub-Saharan Africa [1]. When the incidence of HAT across the continent dropped to such low numbers, the newly-independent nations of sub-Saharan Africa reduced their efforts to monitor and control the disease. This reduction, combined with political and economic turbulence in some of the countries most affected by the disease (e.g., Uganda, Sudan, Angola, Democratic Republic of Congo) led to a resurgence in HAT across the continent, such that by the late 1990s there were >30,000 recorded cases/year. Consequently, the World Health Organization (WHO) revived a major programme of disease surveillance and treatment which has now reduced the annual number of reported cases to <15,000/year [1]. Thus, over the past 80 years, programmes against HAT have been based largely on the detection and treatment of disease in humans and this continues to be the case [1]. Interventions against tsetse flies (*Glossina spp.*) [2], the vector of the *Trypanosoma* spp which cause HAT, have, with some exceptions normally based on the rodesiense form of the disease [3], played a minor role. This emphasis on tackling the trypanosome rather than the tsetse is due to a variety of humanitarian, socio-economic [4],[5],[6] and epidemiological [7],[8] factors. By contrast, tsetse control has played a major role in the control of animal trypanosomiasis [4]. Should vector control play a greater role in tackling HAT?

More than 90% of HAT cases are caused by *T. brucei gambiense* transmitted by Palpalis-group species of tsetse found in Central and West Africa [1]. Moreover, modern methods of tsetse control, based on the use of natural (insecticide-treated cattle) or artificial (traps or insecticide-treated targets) baits to lure and kill tsetse, have the particular advantage that they can be applied and afforded by local people. Such interventions could overcome the present dependence on outside agencies to deploy survey teams and provide drugs and medical personnel. Against this advantage, the application of baits against HAT faces two important problems. First, the use of insecticide-treated cattle [9] depends on cattle being present and forming a significant part of the diet of the local tsetse. In many of the HAT-affected areas of West Africa, cattle are not abundant (e.g., Guinea, southern Côte d'Ivoire, DRC [10] and/or cattle do not seem to be an important component of the diet of Palpalis-group tsetse [11]. Second, the use of artificial baits has been more successful with Morsitans- (e.g., *G. pallidipes* and *G. m. morsitans*) rather than Palpalis-group species of tsetse [12]. Morsitans-group species, especially *G. pallidipes*, are highly responsive to host odours. Insecticide-treated targets and traps, baited with synthetic blends of these odours and deployed at densities of ∼4 targets/km^2^, can eliminate populations of tsetse rapidly [13],[14],[15]. By contrast, there are no artificial attractants effective against important vectors of *T. b. gambiense* and thus baits must be deployed at densities of 30–40 km^−2^ [12] making the method prohibitively expensive [6].

In the period 1997–2006, 92% of the reported ∼242,000 cases of HAT caused by *T. b. gambiense* were in Angola, DRC, Sudan or Uganda. In these countries, either *G. f. fuscipes* (northern DRC, Uganda, Sudan,) or *G. f. quanzensis* (northern Angola, southern DRC) are the only significant vectors [16]. Over the same period (1997–2006), 51% of the ∼6000 reported infections caused by *T. b. rhodesiense* were in southern Uganda where *G. f. fuscipes* is the main vector. Thus, nine out of ten cases of HAT probably start with a bite from a subspecies of *G. fuscipes*. The chemicals used to attract Morsitans-group tsetse are not effective for *G. f. fuscipes* [17]. However, there is evidence that there are novel attractants present in the natural odour from monitor lizard [18],[19], an important host for this species. It has also been reported that oil of *Pinus pumilionis*, octenol and decyl formate is an attractant for these flies [20]. For *G. f. quanzensis*, there are no data beyond Frezil & Carnevale's [21] very limited observation that carbon dioxide increases the catch of tsetse from traps.

As a starting point for any programme to identify attractants, we need to assess whether a species uses odours to locate its hosts. Humans, pigs, cattle and lizards are important hosts for *G. fuscipes* spp [11] but, with the exception of the studies of lizard odours, there are no data to indicate whether these species use odours to locate hosts. Accordingly, this paper reports the results from field studies undertaken in Kenya and the DRC to assess the responses of *G. f. fuscipes* and *G. f. quanzensis*, respectively, to natural odours from humans, cattle, pigs and lizards.

Following the successful approaches used in the identification of attractants for Morsitans-group species [22],[23],[24], we did not confine ourselves to assessing the effect of odours on the catch of tsetse from traps but, rather, used various arrangements of electric nets [25] to quantify the effects of odours on the specific behavioural responses of: (i) long-range attraction, (ii) landing and (iii) trap entry.

## Materials and Methods

### Study sites

#### *G. f. fuscipes*

Studies of *G. f. fuscipes* were undertaken in western Kenya, between July 2007 and December 2008, on the islands of Chamaunga (0°25′S, 34°13′E (grid reference to the Islands location and not to specific trap sites) - ∼0.5 Km^2^ and 500 m from the mainland), Manga (0°21′S, 34°15′E - ∼0.4 Km^2^ and 300 m from the mainland) and the northern peninsula of Rusinga (0°21′S, 34°13′E – essentially part of the mainland to which it is attached by a 100 m causeway) which are all within 5 km of the ICIPE Mbita Point Field station), or at sites on the mainland near Mbita (Kirindo; 0°26′S, 35°15′E) and in Chakol Division of Teso District (0°30–32′N, 34°10–18′E). The islands of Rusinga and Manga are inhabited but Chamaunga is not - apart from occasional visits by fishermen and entomologists. The natural lacustrine vegetation at all these sites has been degraded and fragmented by human activity. Domestic livestock (cattle, sheep, goats), humans and monitor lizards provide the main hosts within the area [26],[27]; wild mammalian hosts, apart from hippopotamus, have been hunted out or driven away by destruction of habitat. For further details of Mbita and its environs, see Mwangelwa *et al.* [28] and Bauer *et al.* [29], respectively.

#### *G. f. quanzensis*

Studies of *G. f. quanzensis* were undertaken in June–August 2008, at sites near the Lukaya river (4°29′S, 15°18′E), ∼20 km south of Kinshasa, DRC. The sites were located on a mixed crop-livestock farm where humans and livestock, particularly pigs, were abundant. No wild hosts of tsetse were seen during the study. For information on tsetse and trypanosomiasis in the area, see De Deken *et al.* [30].

### Natural host odours

In each country, local cattle, pigs or humans were used as sources of host odours (baits). In Kenya only, studies were also made of odours from monitor lizards. The baits were placed in PVC-coated tents (∼2×2×3 m in Kenya; 2×1.5×2 m in DRC) from which the air was exhausted at ∼2000 L/min using a 12 v co-axial fan connected to a flexible PVC-coated tube (0.1 m dia.) with a net-covered outlet placed at ground level, ∼15 m away, where various catching devices were placed. In this way, cattle, humans and pigs were not visible nor could they be bitten by approaching tsetse flies. Lizards were unable to bask in a tent and, being poikilothermic, the absence of basking would reduce their metabolic rate and, perhaps, the odours they produce. Accordingly, they were placed in a chamber (∼2.4×2.4×2.5 m) with stainless-steel walls and a partially shaded glass roof which allowed the lizards contained within it to move freely in and out of shade during the course of an experiment. Studies with Morsitans-group flies suggest that the effectiveness of odours from particular host species is related to their gross weight. Accordingly, to match the weights of different mammalian host species, tents contained a single ox, two men or three-to-four pigs. Given the average weight of the cattle (∼150 kg), humans (∼75 kg) and pigs (∼50 kg) the gross weight of mammalian baits within the tent was 150–200 kg unless reported otherwise. Lizards are considerably smaller and 5–6 lizards (ranging in individual weight from 2.5–7 kg and sex undetermined) with a total, combined weight of ∼30 kg were placed in the tent. Cows and pigs were from local farms and maintained under normal local conditions. Lizards were trapped near the lake when required, held in cages and provided with fish or beef on the evening of every third day and were used in experiments over a period of 12–14 days.

In Kenya only, studies were also made of the responses to urine from lizards collected and dispensed following the methods of Mohamed-Ahmed [18]. Bacterial fermentation of host urine seems to have an effect on their efficacy and so studies were made of the responses to fresh urine and urine that had been fermented for two weeks.

### Synthetic host odours

In some experiments, studies were made of the responses of tsetse to chemicals known to be present in cattle odour and to be effective for some species of tsetse. These chemicals included: acetone (∼500 mg/h), 1-octen-3-ol (octenol; ∼0.1 mg/h), 4-methylphenol (∼0.4 mg/h) and 3-*n*-propylphenol (∼0.01 mg/h) for *G. f. quanzensis* only and carbon dioxide (1–4 L/min) for both. The chemicals were dispensed individually or in various combinations following the methods of Vale & Hall [23] and Torr *et al.* [31].

### Field measurements of carbon dioxide

To measure the dose of carbon dioxide produced by different hosts, the concentration (ppm) of carbon dioxide in the air being exhausted from the tents was measured using an infra-red gas analyser (EGM-1, PP Systems, Hitchin, UK). The velocity of air (m/s) being exhausted from the tent's exhaust pipe was also measured, using a hot wire anemometer, and hence the absolute volume of carbon produced by the test animals could be estimated.

### Catching devices

#### Traps

Biconical [32] or monopyramidal [33] traps were used in Kenya and the DRC, respectively. Standard phthalogen blue (reflectance spectral peak 460 nm – Jenny Lindh, pers. comm.) and black cotton were used throughout.

#### Electric grids

To catch tsetse in flight, electric nets [25] 1 m tall and between 0.5 and 1 m wide, were mounted on a tray (3 cm deep) containing soapy water. The fine black polyester net (Quality no. 166, Swisstulle, Nottingham, UK) and the electrocuting wires of the electric net used here are effectively invisible to tsetse [25],[34]. Flies collided with the electric net and fell, killed or stunned, into the tray where they were retained.

Electric targets were used to catch tsetse as they landed. The targets were similar to the electric nets except that the fine net was replaced by a panel of black cloth. Tsetse were electrocuted as they landed on the target and fell into the water-filled tray. For the analysis, we assumed that the electrified devices killed 100% of the flies contacting them.

In addition to tsetse, the numbers of *Stomoxys* were also recorded. These data are reported where the catches were sufficiently large for analysis and where the results assist in the interpretation of those for tsetse.

### Attraction and landing responses

To assess the numbers of tsetse attracted to various host odours, an electric net (either 0.5 m wide ×1.0 m high or 1×1 m) was placed downwind of the source. Tsetse do not orientate precisely to an odour source unless it is marked by a visual stimulus [22]. Accordingly, a target, consisting of a panel of black cloth (0.75×0.75 m) was placed 0.5 m upwind of the electric net (1×1 m). Alternatively, an electric target (1×1 m or 0.5 m high×1 m wide) was placed adjacent to the smaller (0.5 m wide×1 m high) electric net. For experiments where an electric net and electric target were used in combination, the catch from the target, expressed as a proportion of the total (i.e., net+target) catch, provided an index of the strength of the landing response. Henceforth, an electric net operated singly is referred to as an ‘E-net’ and the combination of an electric target+flanking electric target is termed an ‘E-target’.

### Trap-oriented responses

To assess the effect of host odours on trap-oriented responses, odours were dispensed at the base of the trap. The catch from a trap is the product of (i) the number of tsetse attracted to the vicinity of the trap and (ii) the proportion that subsequently enter it and are retained – i.e., the so-called ‘trap efficiency’ [35]. Odours can have effects on attraction and/or trap efficiency. To measure these effects independently, experiments were performed with an electric net (0.5 m wide×1 m high) placed adjacent to the trap. The total catch (electric net+trap) provided a measure of the numbers of tsetse attracted to the trap with or without host odours, and the catch from the trap, expressed as a proportion of the total catch, provided an index of trap efficiency.

### Experimental design and analyses

All field experiments were carried out for 4 h between 08:00 h and 14:00 h local time when Palpalis-group species are most active [26],[36]. In general, odour baited devices (e.g., traps, electric nets, electric targets and combinations thereof) were compared with an unbaited device over 6–12 days in a series of replicated Latin squares of days×sites×treatments. Sites were between 100 m and 200 m from each other.

The daily catches (*n*) were normalized and variances homogenized using a log_10_(*n*+1) transformation and then subjected to analysis of variance using GLIM4 [37]. In general, the detransormed means are reported accompanied by their transformed means and standard errors of the difference (SED) between means [37]. To provide a common index of the effect of odours on catches, the detransformed mean catch of tsetse from an odour-baited device was expressed as the proportion of that from an unbaited one. The value is termed the catch index; odours which, say, double or halve the catch from a trap would have catch indices of 2 and 0.5, respectively.

Logistic regression was used to analyse the effects of odours on the proportions that were caught landing on a target or entering a trap as opposed to colliding with a flanking electric net. Following Crawley [37], the total daily catches from a particular device (e.g., target+flanking net, trap+flanking net) were specified as the binomial denominator and the catches from the accompanying target or trap as the y-variable. Days, sites and treatments were specified as factors and the statistical significance of differences in the proportion of tsetse landing on the target or entering a trap was assessed by removing the treatments factor from the full model (i.e., days+sites+treatments). The significance of changes in deviance was assessed by either χ^2^ or, if the data were overdispersed, an *F*-test following re-scaling [37]. The SE is asymmetric about the mean and thus mean percentages are accompanied by the larger SE. For all analyses, the term “significant” implies *P*<0.05.

## Results

### Attraction to natural host odours

#### *G. f. fuscipes*

The results for *G. f. fuscipes* (Table 1) show that odour from cattle, humans and pigs (Experiments 1–8) had no effect on the numbers of tsetse caught by a trap or electrocuting device. The geometric mean of the catch indices for cattle, human and pig odour were 1.04, 1.08 and 1.25, with only pig odour in one experiment having a significant effect for males. By contrast, odours from lizards increased the catch of males and females significantly in four out of five experiments. There is an absence of a consistent and significant effect for mammalian odours despite the fact that carbon dioxide dispensed outside the tent from a cylinder at 2 L/min increased the catch 1.4×, with the increase being significant for females (Table 1, Experiment 12). Four cattle, with a combined weight of ∼600 kg, would be expected to produce carbon dioxide at >2 L/min [38] and yet natural ox odour from this source (Table 1, Experiment 8) had no significant effect. In addition, when carbon dioxide was dispensed from within a tent there was not a significant increase (Table 1, Experiment 13). The absence of any effect for mammalian odours and the effect of lizards does not seem to be associated with particular locations. For instance, the catch indices for females at an E-net baited with ox- or lizard-odour were 1.4 and 2.1, respectively on Chamaunga island (Table 1, Experiment 5) compared to 1.3 and 3.0 for Teso (Experiments 4 and 9). Chamaunga is a small uninhabited island where monitor lizards are abundant whereas Teso is a settled area with a wide range of potential mammalian hosts, including cattle and humans [27].

**Table 1:** Detransformed mean daily catches (transformed mean and standard error of the difference (SED) shown in brackets) of *G. f. fuscipes* caught over *n* days from odour-baited devices at various locations in western Kenya.

Odour	Device	Location	Expt	Days	Males			Females		
					Catch	(m±sed)	Index	Catch	(m±sed)	Index
Cattle (×1)	E-net	Manga	1	12	17.4	(1.27±0.084)	**1.1**	25.0	(1.42±0.057)	**1.1**
	E-net	Manga	2	8	7.5	(0.93±0.127)	**1.3**	24.2	(1.40±0.069)	**1.4**
	E-target	Rusinga	3	8	8.3	(0.97±0.068)	**0.9**	21.5	(1.35±0.065)	**0.9**
		Teso	4	12	1.8	(0.25±0.052)	**1.3**	4.0	(0.60±0.066)	**1.1**
	E-target (S)	Chamaunga	5	12	14.2	(1.18±0.069)	**1.2**	5.8	(0.83±0.093)	**1.4**
	Trap	Chamaunga	6	8	2.9	(0.59±0.145)	**0.9**	2.5	(0.55±0.114)	**0.6**
	Trap+E-net	Chamaunga	7	12	6.9	(0.90±0.057)	**1.1**	7.4	(0.93±0.073)	**0.8**
Cattle (×4)	E-target	Rusinga	8	10	21.3	(1.35±0.062)	**1.0**	41.3	(1.63±0.057)	**1.1**
Humans (×2)	E-Net	Manga	1	12	14.5	(1.19±0.084)	**0.9**	20.1	(1.32±0.057)	**0.9**
	E-Net	Manga	2	8	5.7	(0.83±0.127)	**1.0**	21.9	(1.36±0.069)	**1.2**
	E-target	Rusinga	3	8	10.1	(1.05±0.068)	**1.0**	26.9	(1.45±0.065)	**1.1**
		Teso	4	12	1.8	(0.26±0.052)	**1.3**	4.2	(0.62±0.066)	**1.2**
	E-target (S)	Chamaunga	5	12	14.1	(1.18±0.069)	**1.2**	7.3	(0.92±0.093)	**1.3**
	Trap	Chamaunga	6	8	3.7	(0.67±0.145)	**1.1**	4.2	(0.71±0.114)	**1.0**
	Trap+E-net	Chamaunga	7	12	6.7	(0.89±0.057)	**1.0**	7.9	(0.95±0.073)	**0.9**
Pigs (×2–3)	E-Net	Manga	1	12	14.1	(1.18±0.084)	**0.9**	18.6	(1.29±0.057)	**0.8**
		Manga	2	8	8.8	(0.99±0.127)	**1.5**	28.0	(1.46±0.069)	**1.6**
	E-target	Rusinga	3	8	11.3	(1.09±0.068)	**1.2**	25.2	(1.42±0.065)	**1.1**
		Teso	4	12	2.4	(0.38±0.052)	1.8*	4.2	(0.63±0.066)	**1.2**
	Trap	Chamaunga	6	8	5.1	(0.79±0.145)	**1.6**	5.6	(0.82±0.114)	**1.3**
Lizards (×6)	Trap	Teso	9	12	3.0	(0.60±0.097)	2.6*	9.9	(1.04±0.040)	3.0***
	E-target (S)	Rusinga	10	12	28.2	(1.47±0.051)	**1.2**	61.2	(1.79±0.040)	**1.2**
		Chamaunga	5	12	18.3	(1.29±0.069)	1.5*	12.3	(1.12±0.093)	2.1*
	Trap+E-net	Rusinga	11	12	41.1	(1.62±0.048)	1.4*	66.6	(1.83±0.034)	1.5***
		Chamaunga	7	12	14.2	(1.18±0.057)	2.2***	14.2	(1.18±0.073)	1.5**
CO_2_ (2 L/min) – out	E-target	Rusinga	12	6	26.0	(1.43±0.028)	**1.2**	53.6	(1.74±0.024)	1.6*
		Kirindo	13	9	10.6	(1.06±0.103)	2.9**	20.6	(1.33±0.081)	2.1**
CO_2_ (2 L/min) – in		Kirindo	13	9	6.3	(0.87±0.103)	**1.8**	14.8	(1.2±0.081)	**1.5**

Carbon dioxide was dispensed within (‘in’) or outside (‘out’) the tent. The detransformed mean daily catch of each odour-baited devices is expressed as a proportion (Index) of that from an unbaited device; asterisks indicate that the index is significantly different from unity at the P<0.05 (*), P<0.01 (**) or P<0.001 (***) levels of probability. Treatments with the same experiment number (Expt.) were incorporated into the same Latin square.

Baiting traps with fresh or fermented lizard urine had no significant effect on the catch. Traps baited with fresh urine caught 14 (1.18±0.053, detransformed mean±SED) males and 20 (1.31±0.038) females per day compared to 16 (1.22±0.053) males/day and 19(1.31±0.038) females/day from an unbaited trap. Traps baited with fermented urine caught 10 (1.04±0.062) males and 15 (1.20±.051) females per day compared to 10 (1.03±0.062) males/day and 13 (1.150±.051) females/day from an unbaited trap.

Three experiments were performed when *Stomoxys* was abundant and analysis of the results showed that the absence of any response by *G. f. fuscipes* to cattle odour was not due to, say, a malfunction of the sampling devices or some other experimental artefact. First, studies using a trap+E-net conducted on Chamaunga showed that cattle and human odour had no significant effect on the catches of tsetse (Table 1, Experiment 7) but lizard odour increased the catch of male and female *G. f. fuscipes* by 2.2× and 1.5×, respectively. For *Stomoxys*, cattle odour increased (*P*<0.001) the catch 10× whereas human and lizard odours had no significant effects; the cattle-baited trap+E-net caught 98 (2.0±0.101, detransformed mean±SED) *Stomoxys*/day compared to 8 *Stomoxys*/day with lizard odour (0.99±0.101) and 9 *Stomoxys*/day for the human- (1.00±0.101) and unbaited (1.00±0.101) traps. Second, baiting a small E-target with lizard odour increased the catch of tsetse whereas cattle and human odour had no significant effect (Table 1, Experiment 5). The catches of *Stomoxys* from lizard-, human-, and ox-baited E-nets were 6 (0.82±0.122) 4 (0.72±0.122) and 37 (1.58±0.122) compared to 5 (0.79±0.122) from an unbaited E-target. Third, baiting an E-target with the odour from four cattle had no significant effect for *G. f. fuscipes* (Table 1, Experiment 8) but increased the catch of *Stomoxys* significantly from 4 (0.71±0.102) *Stomoxys*/day for an unbaited trap to 17.5 (1.27±0.102) Stomoxys/day for one baited with ox odour.

#### *G. f. quanzensis*

The data for *G. f. quanzensis* (Table 2) also show that human and cattle odours had no significant effect on the catch, but pig odour increased the catch of females from odour-baited targets significantly. Analysis of the pooled data from the 24 days when the odour from three pigs was tested showed that there was a highly significant (*P*<0.001) doubling in the catch of females from 2.3 (0.51±0.069) to 4.8 (0.76 0.081) per day whereas there was no significant effect for males, i.e., 2.9 (0.59±0.076) per day with pig odour *vs.* 2.6 (0.55±0.089) without odour. Carbon dioxide dispensed at 1–2 L/min within a tent also increased the catch of tsetse, with the increase being greater for females than males. The effect of natural pig odour might be explained, in part, by carbon dioxide produced by the pigs. Accordingly, direct comparisons were made of the numbers of tsetse attracted to a target baited with either the pig odour or an equivalent dose of carbon dioxide. In one experiment where the odour from three pigs was compared with carbon dioxide dispensed at 1 L/min, more tsetse were caught by the target baited with carbon dioxide than the pig-baited one (e.g., 4.3 females/day *vs.* 3.8 females/day; see Table 2, Experiment 2) and in a second experiment the target baited with the odour from seven pigs caught slightly more tsetse than a target baited with carbon dioxide dispensed at 2 L/min (eg, 6.1 females/day *vs.* 4.4 females/day; see Table 2, Experiment 4). In neither experiment was there a significant difference in the catch from the pig- and CO_2_-baited E-targets but both were significantly greater than that from an unbaited E-target. However, the humans and cattle also produced equivalent doses of carbon dioxide and yet these failed to elicit a significant increase in catch index.

**Table 2:** Detransformed mean daily catches (transformed mean and standard error of the difference (SED) shown in brackets) of *G. f. quanzensis* from odour-baited devices operated in five experiments conducted in the Democratic Republic of Congo.

Odour	Device	Expt.	Days	Males			Females		
				Catch	(m±sed)	Index	Catch	(m±sed)	Index
Pig (×3)	E-target	1	12	3.5	(0.65±0.107)	**1.1**	6.1	(0.85±0.096)	2.4*
		2	12	2.4	(0.54±0.080)	**1.2**	3.8	(0.68±0.087)	1.9*
	Trap	3	4	0.9	(0.27±0.158)	**0.7**	0.3	(0.12±0.080)	**0.6**
Pig (×7)	E-target	4	12	3.1	(0.61±0.091)	**1.2**	6.1	(0.85±0.088)	3.0**
Human (×2)	E-target	1	12	4.6	(0.75±0.107)	**1.4**	3.2	(0.62±0.096)	**1.3**
		2	12	2.7	(0.56±0.080)	**1.3**	3.1	(0.61±0.087)	**1.5**
	Trap	3	4	0.9	(0.27±0.158)	**0.7**	0.4	(0.15±0.080)	**0.7**
Cattle (×1)	E-target	1	12	3.3	(0.64±0.107)	**1.0**	3.6	(0.67±0.096)	**1.4**
	Trap	3	4	2.2	(0.51±0.158)	**1.8**	1.0	(0.30±0.080)	**1.8**
CO_2_ (1 L/min) – in	E-target	2	12	2.9	(0.59±0.080)	**1.4**	4.3	(0.72±0.087)	2.1*
CO_2_ (2 L/min) – in	E-target	4	12	5.0	(0.78±0.091)	**1.9**	4.4	(0.73±0.088)	2.1*
AOP	Trap	5	12	0.8	(0.26±0.111)	**0.8**	0.9	(0.28±0.100)	**1.0**

Carbon dioxide was dispensed within (‘in’) a tent only. The detransformed mean daily catch (Catch) of each odour-baited devices is expressed as a proportion (Index) of that from an unbaited device. Asterisks indicate that the index is significantly different from unity at the P<0.05 (*) or P<0.01 (**) levels of probability. Treatments with the same experiment number (Expt.) were incorporated into the same Latin square.

Baiting traps with natural odours or a blend of acetone, octenol and phenols (AOP) had no significant effect on the catch of *G. f. quanzensis* (Table 2).

### Landing responses

**Figure 1:**
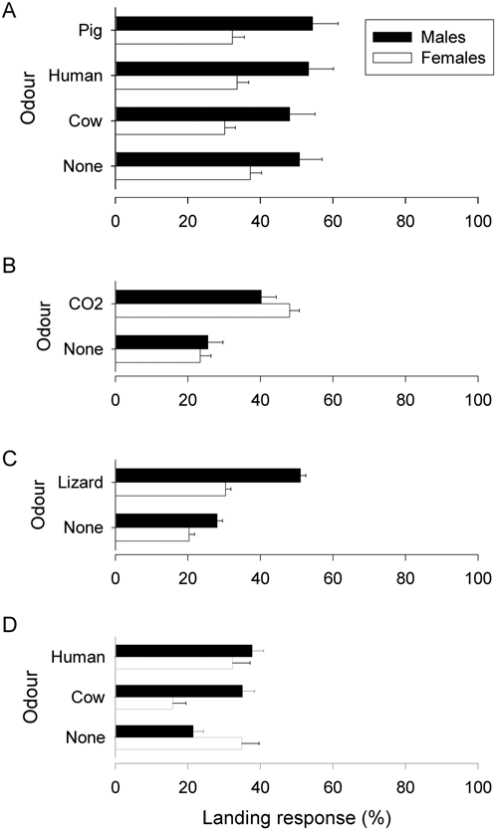
Landing response of *G. f. fuscipes* on a large (1×1 m) E-target baited with either (A) mammalian host odours or (B) carbon dioxide dispensed outside a tent, or on a small (0.5 m high×1 m wide) E-target baited with (C) lizard or (D) mammalian host odours. The landing response is the number of tsetse caught landing on the target expressed as a percentage of the total (landing+circling) catch.

The results for *G. f. fuscipes* (Fig. 1) show that odours from humans, cattle and pigs had no significant effect on the proportion of tsetse that were caught as they landed on the cloth panel of the large (1×1 m) E-target (Fig. 1A): for all treatments, ∼30% of males and ∼50% of females landed on the target. In one experiment (Experiment 12), carbon dioxide dispensed outside a tent increased significantly the proportion of female *G. f. fuscipes* that landed on the target (Fig. 1B) (48% *vs.* 23%) and had a similar effect, albeit not statistically significant, for males (40% vs. 26%). In a second experiment (Experiment 13) where the effect of dispensing carbon dioxide inside or outside a tent was assessed, there was no significant effect, although the trend was similar: 43% (±3.5) of females landed when carbon dioxide was dispensed outside, 34% (±3.8) when it was dispensed inside and 30% (±4.4) for an unbaited target. These results from Experiment 13 also suggest that carbon dioxide was more effective when dispensed outside a tent (landing response = 40–48%) than within (34%) which is consistent with the indications (above) that carbon dioxide attracted more tsetse when it was dispensed outside a tent rather than within it. Baiting a small (1.0 m wide×0.5 m high) target with lizard odour increased the landing response for males and females significantly (Fig. 1C). Baiting the small target with mammalian host odours also had a significant effect: human and cattle odour increased the landing response for males significantly whereas cattle odour *decreased* the response for females significantly.

**Figure 2:**
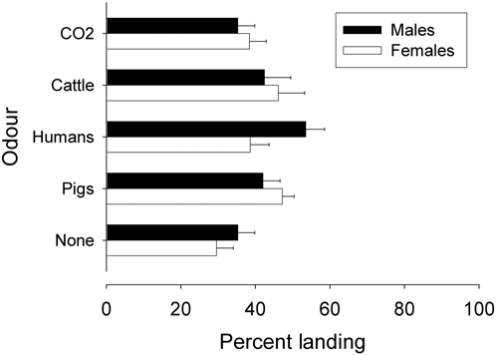
Landing response of *G. f. quanzensis* on an E-target baited with natural host odours or carbon dioxide dispensed inside a tent. The landing response is the number of tsetse caught landing on the target expressed as a percentage of the total (landing+circling) catch.

The mean daily catches of *G. f. quanzensis* from an E-target were much smaller than the catches of *G. f. fuscipes* in Kenya; the geometric mean of the total (males+females) daily catches of *G. f. fuscipes* shown in Table 1 is 23 tsetse/day compared to 5 tsetse/day for the catches of *G. f. quanzensis* shown in Table 2. The small daily catches of *G. f. quanzensis* prevented analysis of landing rates from individual experiments. Accordingly, the data from all experiments were pooled and subjected to logistic regression. The results (Fig. 2) show that there was no significant effect of host odours on the landing response. However, the landing rate of females was consistently higher in the presence of pig odours; in the three experiments where pig-baited and unbaited E-targets were compared directly, the landing rates with pig odour were 43% (*n* = 176), 46% (*n* = 156) and 52% (*n* = 84) compared to 19% (*n* = 86), 35% (*n* = 68) and 37% (*n* = 38), respectively, for an unbaited E-target. By contrast, there was no indication that carbon dioxide increased the landing rate.

Experiments conducted when *Stomoxys* was abundant showed that cattle odour increased the landing response significantly. For instance, the landing response of *Stomoxys* on a small E-target baited with cattle (58±3.0%) was significantly greater than that from lizard- (37±7.6%), human- (38±8.8%) or unbaited (31±7.8%) E-targets. Baiting a large E-net with odour from four cattle increased the landing response significantly from 21±9.8% to 55±4.9%.

### Trap efficiency

**Figure 3:**
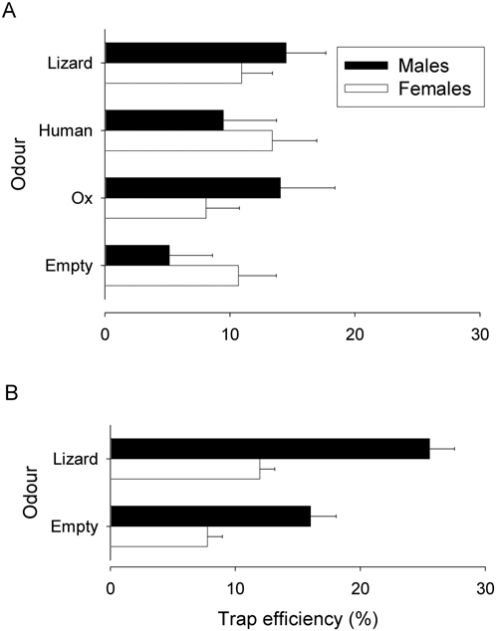
Effect of mammalian (A) and lizard (A & B) host odours on trap efficiency. Trap efficiency was gauged by expressing the mean catch of *G. f. fuscipes* caught in a trap alone as a percentage of the total catch from the trap+flanking E-net.

Studies of the effect of odours on trap efficiency were made for *G. f. fuscipes* only. The results (Fig. 3) show that in one experiment (Fig. 3A) conducted on Chamaunga, host odours had no significant effect for males or females. In a second experiment (Fig. 3B), lizard odour increased the percentage of males and females entering the trap. The difference in effects for lizard odour may merely reflect differences in the sample sizes which allowed us to detect relatively small (∼10%) increases in trap efficiency. The total catches of males and females from the lizard-baited trap for experiment A, where no statistically significant effects were apparent, were 207 and 192, respectively, compared to 505 and 811 for experiment B. Host odours had no significant effect on trap efficiency for *Stomoxys*.

## Discussion

The present results confirm the previous indications that Palpalis-group tsetse are markedly different to the Morsitans-group in their responses to host odours in several respects.

### Attraction to sources of mammalian host odours

Baiting an E-target with odours from cattle, human or pig odours had no significant effect for *G. f. fuscipes* and only pig odour increased the catch significantly for *G. f. quanzensis*. For Morsitans-group species by contrast, cattle odour increases the catch ten-fold [22],[39]; pig (warthog and bushpig) odours are also highly effective [22], and human odour seems to contain a mixture of attractants and repellents [35].

The present experiments were performed at a variety of sites with various sampling devices and host animals and hence it seems unlikely that the absence of any marked response is an experimental artefact. Moreover, the absence of any response to cattle odour is consistent with previous studies showing that cattle kairomones effective for Morsitans-group tsetse (i.e., acetone, octenol and phenols) are ineffective for *G. f. fuscipes* [17]. The present results show that these odours are also ineffective for *G. f. quanzensis*.

### Responses to carbon dioxide

Carbon dioxide is present in the odours produced by all living hosts and is commonly claimed to be a universal kairomone for biting flies, including tsetse. Carbon dioxide dispensed alone, doubles the catch of both sexes of *G. m. morsitans* and *G. pallidipes* [22] and acts synergistically with other host kairomones [40]. Thus it is surprising that the natural odours that contained this gas were ineffective for *G. f. fuscipes* whereas carbon dioxide dispensed alone, at 2 L/min, did have a significant effect, albeit slight (2×) and only when the gas was dispensed outside the tent. Dispensing carbon dioxide within a tent will dilute the concentration of the odour at the source; the 2 L/min of gas released is diluted in ∼2000 L of air being exhausted from the tent giving a source concentration of ∼0.1% compared to 100% at the point where carbon dioxide is released from a tube connected to a gas cylinder outside the tent. However, for Morsitans-group species at least, this difference does not seem to affect catches significantly [22],[41], probably because the diluting effects of atmospheric turbulence on the odour plume as it travels downwind, obscures the differences in source concentration [42].

*G. f. quanzensis* was responsive to carbon dioxide dispensed within a tent, but the increase was relatively slight (∼2×) and only significant for females. Natural host odours that included carbon dioxide (e.g., human odour) were not, however, consistently effective and *G. f. quanzensis* also seems to display only a slight and variable response to carbon dioxide.

These results accord with those of Mohamed-Ahmed & Mihok [43] who also reported variable responses of *G. f. fuscipes* to carbon dioxide. They baited traps with carbon dioxide dispensed directly from a concealed cylinder placed nearby (i.e., the gas was dispensed outside a tent). In one experiment they found that carbon dioxide dispensed at 5 L/min had no significant effect whereas in a second experiment, with the carbon dioxide dispensed at a lower dose of 2.5 L/min, the catch of females, but not males, was doubled.

*Stomoxys* is considered to be highly responsive to carbon dioxide [23],[38]. Given that all host odours contain carbon dioxide, it is surprising that, in the present study, cattle odour was highly effective for *Stomoxys* whereas pig and human odours were not. Thus for the populations of *Stomoxys* studied here, the olfactory response to cattle odour seems to be elicited by kairomone(s) other than carbon dioxide, whereas studies conducted elsewhere suggest that carbon dioxide is the major kairomone that attracts *Stomoxys* produced by cattle [23],[38],[44]. It is therefore remarkable that in this part of western Kenya, carbon dioxide is not as effective as expected for two genera of biting flies.

### Responses of *G. f. fuscipes* to lizard odours

While *G. fuscipes* spp. seem unresponsive to mammalian odours, the present results show that there is a clear and consistent response to natural lizard odour, according with the findings of Gouteux [19] and Mohamed-Ahmed [18]. However, in the present study, lizard urine had no significant effect whereas Mohamed-Ahmed [18] found that urine doubled the catch of female *G. f. fuscipes* attracted to an electrocuting cylinder and increased the catch of tsetse from a trap 1.5×. However even his results are marginal: the increase with the electrocuting cylinder are not significant for either males or females analysed separately, and the increase with traps is only significant for males. Mwangelwa *et al.* [17] found that aqueous washings of monitor lizard had no significant effect on the catch of a trap. However, given that monitor lizards are semi-aquatic, it would be surprising if tsetse evolved responses to odours that could be readily washed off.

The effect of lizard odour is unlikely to be explained by a response to carbon dioxide as the lizard biomass in the tents was only 20% of the mamalian hosts. Accordingly lizards increased the concentration of carbon dioxide by only ∼100 ppm above background, compared to 2000 ppm above background for a carbon dioxide dispensed at 2 L/min within a tent. Since the latter did not have a significant effect, and that carbon dioxide is more effective at higher concentration, it seems unlikely that the small amount of carbon dioxide produced by lizards accounts for their attractiveness.

### Less responsive, unresponsive or just different?

The present results show that while *G. f. fuscipes* is responsive to lizard odour the fly does not respond to odour in the same way as Morsitans-group species. We carried out experiments that can produce large effects for Morsitans-group species, but perhaps these experiments are not appropriate for the particular host-location strategies of Palpalis-group species. Indeed, the small hosts (e.g., lizards) and dense vegetation that often characterises the ecology of riverine flies might lead us to expect that strategies based on responses to olfactory cues would be particularly advantageous. By contrast, the large hosts (e.g., buffalo, antelope, warthog) and open savannah habitats typical of Morsitans-group species suggests that visual cues should be more important.

The paradigm for the odour-orientated behaviour of ‘tsetse’ is based largely on the responses of *G. pallidipes*. For this species, the large catches produced by odours arise because tsetse are recruited to the source from distances of up to 100 m by upwind anemotaxis (see review by [45] and references therein). However, several factors might suggest that this paradigm does not apply to Palpalis group species. First, the variable responses to carbon dioxide obtained by Mohamed-Ahmed & Mihok [43] were attributed to the linear nature of the habitat: carbon dioxide was ineffective in a ‘linear forest’ because the odour plume extended into areas outside the forest where tsetse were absent. While this might limit responses to host odours in some situations, we do not think that this is a universal explanation for the unresponsiveness of Palpalis-group flies. We carried out the experiments in a variety of habitats, where the distribution did not appear to be markedly linear, and yet mammalian odours were always ineffective for *G. f. fuscipes*. Second, studies of the odour-orientated responses of Morsitans-group flies have shown that low wind speeds caused, for example, by dense vegetation acting as a windbreak, can reduce the effectiveness of host odours [46]. Moreover, in densely vegetated habitats with low wind speeds and high photosynthetic activity, the background noise of atmospheric carbon dioxide can be 10× greater than that observed in dry savannah woodland [42]. Both these factors would limit the effective range of host odour plumes, especially those that relied on anemotactic responses to carbon dioxide. Mohamed-Ahmed & Mihok's [43] finding that carbon dioxide was effective in one experiment but not another may not have been due to the topography of the forest (see above) but, rather, the time of year when the experiments were performed. They found that carbon dioxide was effective in the dry season but not in the wet. Work conducted in southern Africa suggests that the background noise of atmospheric carbon dioxide is higher during the wet season. At the field sites in Kenya, atmospheric carbon dioxide levels will be affected by the lake, and high resolution measurements of carbon dioxide [42] would be required to test this hypothesis. Ironically, it may be that the places where host odours might be most useful are also those where carbon dioxide produced by hosts is harder to detect and track.

If host odours do not elicit long-range anemotaxis in Palpalis-group flies, might they play other roles? The observation that carbon dioxide is effective whereas natural host odours containing equivalent doses of carbon dioxide are not, might suggest that the host odours contain repellents. The apparent reduction in the landing response of female *G. f. fuscipes* on small targets is also in accordance with this notion.

Perhaps therefore, Palpalis flies do make important use of odours but in a distinctive strategy that we have yet to discern. Might, for instance, odours have orthokinetic or orthotactic effects? In the course of conducting the experiments in Kenya, we frequently observed tsetse resting on the ground near the host for extended periods; behaviour that we have not seen during our studies of Morsitans tsetse. Other studies have reported that lizard urine is effective [18] – might the residues of lizards cause tsetse to congregate in areas where lizards are common, such as basking points along the lake shore?

Discerning the behavioural basis of these effects is important in two respects: first, understanding the effects of odours will allow us to design more sensitive bioassays of putative kairomones and, second, show how to develop strategies to make best use of these odours to control and monitor tsetse.

In conclusion, the present findings suggest that unidentified chemicals present in lizard odour can double the numbers of *G. f. fuscipes* attracted to traps or killed by insecticide-treated targets. And the results for *G. f. quanzensis* suggest that pig odour contains chemicals that increase the landing response and hence the performance of targets. The present results, and experience with other species of tsetse (e.g., [47]), further suggest that larger doses of host kairomones produce larger catches of tsetse. Accordingly, we might reasonably expect that super-normal doses of synthetic attractants will produce even greater improvements in the efficacy of baits for controlling vectors of HAT. But if these improvements are to be realised, or even exceeded, we need to increase our understanding of the specific behavioural effects of these novel odours.
